# Self-Assembled Rg3/Naringenin Nanoparticles for Targeted Brain Delivery: A Promising Therapeutic Approach for Early Alzheimer’s Disease

**DOI:** 10.3390/ph19030367

**Published:** 2026-02-26

**Authors:** Xinru Lou, Zhaolan Ni, Shuning Cui, Zhongmei He, Ying Zong, Weijia Chen, Jianan Geng, Jia Zhou, Zhuo Li, Yan Zhao, Hongbo Teng

**Affiliations:** 1 College of Chinese Medicinal Materials, Jilin Agricultural University, Changchun 130118, China; 13230315313@163.com (X.L.); nizl2024@163.com (Z.N.); 18943010080@163.com (S.C.); heather78@126.com (Z.H.); zongying7699@126.com (Y.Z.); chenweijia-jlau@163.com (W.C.); gengjianan@jlau.edu.cn (J.G.); 007152@jlau.edu.cn (J.Z.); lizhuo@jlau.edu.cn (Z.L.); 2 International Joint Laboratory for Development of Animal and Plant Resources for Food and Medicine, Jilin Agricultural University, Changchun 130118, China

**Keywords:** Alzheimer’s disease, ERK signaling pathway, Fos, ginsenoside Rg3, naringenin, oxytocin

## Abstract

**Background/Objectives**: Alzheimer’s disease (AD) has an irreversible disease course, making early intervention a key measure to delay disease progression. However, existing therapies are limited by weak brain-targeted delivery efficiency due to the blood–brain barrier (BBB) and low bioavailability of drugs, making it difficult to address the complexity of AD’s pathological mechanisms. **Methods**: Addressing these limiting factors, this research aims to develop an early AD intervention formulation with “high targeting, high bioavailability, and high biosafety.” Based on the principle of drug synergistic effects, this study employed the reverse solvent method and optimized the combination ratio of Ginsenoside Rg3 and Naringenin (Nar) to design and prepare a self-assembling nano-delivery system (Rg3-Nar-NPs, GNN). The study utilized intranasal administration to bypass the BBB through the direct pathway between the nasal mucosa and central nervous system. **Results**: This approach enabled targeted accumulation of the drug in brain lesion areas, significantly reducing Aβ deposition, oxidative stress, and inflammatory factor surges caused by early AD, thereby improving cognitive dysfunction in mice. Moreover, GNN demonstrated superior biosafety and bioavailability compared to the individual components. Through transcriptomic analysis, the study elucidated for the first time that GNN can activate the OXT/ERK/Fos pathway to break the malignant cycle of ROS–neuroinflammation, inhibiting the amplification effect of early AD pathological damage. **Conclusions**: This research provides new molecular targets and drug options for multi-target synergistic intervention of early AD, showing potential as a candidate strategy for precise early AD intervention and laying theoretical and experimental foundations for subsequent clinical translation.

## 1. Introduction

Alzheimer’s disease (AD) has been identified as the most prevalent form of dementia; the principal manifestations are memory impairment and functional deterioration. The global incidence of AD exhibits an alarming upward trajectory that correlates directly with demographic shifts toward population aging, presenting an escalating healthcare challenge with profound socioeconomic implications [1]. The pathophysiology of AD manifests according to multiple interconnected mechanisms; the primary manifestations include β-amyloid (Aβ) deposition, tau hyperphosphorylation, cholinergic neuronal degeneration, oxidative stress, and neuroinflammation. Within this pathological framework, the aberrant accumulation of Aβ_1–42_ oligomers represents a critical mediator in neuronal dysfunction and degeneration [2,3].

Studies show that reactive oxygen species (ROS) and brain inflammation are key drivers of AD, with a complex vicious cycle mechanism existing between these two factors [4]. Specifically, Aβ deposition promotes free radical generation, thereby inducing oxidative stress and subsequently precipitating cellular damage and stress responses [5]. Oxidative damage activates immune cells including microglia and astrocytes, promoting the release of inflammatory mediators and subsequently inducing neuroinflammation. Conversely, inflammatory factors released during this inflammatory response further exacerbate oxidative stress. This bidirectional interaction demonstrates that oxidative stress and inflammation mutually reinforce each other, progressively establishing a “ROS-Neuroinflammation Vicious Cycle” [6]. This cyclical mechanism perpetually amplifies cellular damage, accelerating disease progression. Current clinical therapeutic strategies, including cholinesterase inhibitors and neurotransmitter receptor modulators, merely delay disease progression without addressing the fundamental pathology, thus failing to provide curative intervention for AD [7].

Significant potential has been demonstrated by traditional Chinese medicine in the field of neuroprotection. Within the traditional Chinese medicine (TCM) theoretical framework, dementia is primarily attributed to cerebral malnutrition, spleen–kidney deficiency, blood stasis, and phlegm retention [8]. Ginseng and dried tangerine peel are traditionally used to treat cognitive disorders, known as “dai bing” [9]. Research has demonstrated that ginsenoside Rg3, a primary active component of ginseng, possesses antioxidant, anti-inflammatory, and neuroprotective properties [10]. Multiple studies have demonstrated that Rg3 can mitigate the pathological progression of neurodegenerative diseases [11]. Similarly, naringenin, a natural flavonoid compound in dried tangerine peel, exhibits significant antioxidant and anti-inflammatory properties, demonstrating neuroprotective potential across various central nervous system disease models [12]. These two natural compounds have limited clinical applications because of their low bioavailability and poor blood–brain barrier (BBB) permeability [13].

Nanotechnology provides new strategies for the development of drug delivery systems to address the challenges [14]. Nano-delivery systems, with their small particle size and large specific surface area, not only enhance drug solubility and brain distribution but also effectively protect drugs from enzymatic degradation. Self-assembling nanoparticles, due to their simple preparation, high drug loading capacity, and excellent biocompatibility, have become ideal neuroprotective drug delivery platforms that promote cellular uptake and increase intracellular drug concentrations [15,16]. A new strategy that bypasses the BBB through olfactory and trigeminal nerve pathways is provided by intranasal administration combined with nanotechnology, delivering drugs directly to the central nervous system. This approach enhances brain drug utilization and targeting while reducing systemic adverse reactions, opening new avenues for tackling AD [17].

The self-assembled GNN nanostructure is strategically designed for intranasal delivery by providing dual protective mechanisms [18]. First, physical encapsulation of Rg3 and naringenin within the nanoparticle matrix shields them from nasal proteases and esterases, reducing enzymatic degradation and improving compound stability [19]. Second, self-assembled nanoparticles prolong residence time at the nasal mucosa through van der Waals interactions and hydrophobic effects with mucosal components [20]. This combined strategy maximizes bioavailability and nose-to-brain transport, thereby enhancing therapeutic efficacy for early Alzheimer’s disease treatment.

Oxytocin (OXT), as a neuropeptide, exerts neuroprotective effects by regulating the ERK/Fos signaling pathway [21]. It is formed by nine amino acids. It exerts various effects. These are mainly performed via oxytocin receptor (OXTR) activity, present all around the hippocampus—parts of the brain associated with thinking and remembering—and are important for both cognitive functions and brain plasticity [22]. The Erk, or ERK, pathway is involved in keeping neurons alive, cell survival, and learning and remembering [23]. Meanwhile, Fos is a very quick gene, which indicates that neurons are active, and is also involved in learning and remembering [24]. After phosphorylation, ERK1/2 can activate transcription factor Fos to form the AP-1 complex with JUN Family cells. The AP-1 complex is involved in inflammation response and cell apoptotic processes [25]. The inhibited ERK/FOS pathway can reduce levels of inflammation and apoptosis [26]. However, the regulatory mechanism of the ERK/FOS pathway in the early stages of AD and its relationship with drug-mediated neuroprotection have not yet been fully elucidated.

Based on this background, this research aims to develop ginsenoside Rg3 and naringenin self-assembling nanoparticles (GNNs), unlike previous studies that examined Rg3 or naringenin individually, this study is the first to investigate their synergistic co-delivery within a single self-assembled nanoplatform for brain-targeted intranasal administration, thereby achieving multi-pathway neuroprotection, including Aβ inhibition, neuroinflammation reduction, and oxidative stress mitigation simultaneously [27]. Therefore, we evaluate their therapeutic effect on early AD mouse models induced by Aβ_1–42_ oligomers, and elucidate the neuroprotective mechanisms mediated by GNNs, providing important evidence for AD treatment.

## 2. Results

### 2.1. Simulation, Preparation, and Characterization of GNNs

**Figure 1:**
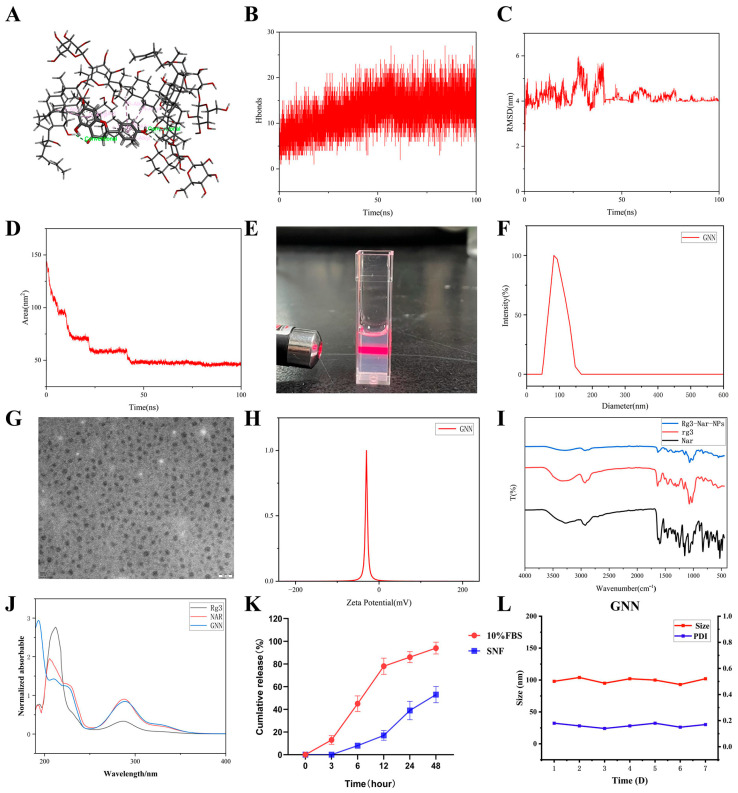
Simulation and characterization of GNNs. (**A**) Interaction mode between NAR and Rg3 (green: Hydrogen bond; pink: Pi–Alkyl). (**B**) Number of hydrogen bonds after NAR and Rg3 binding. (**C**) RMSD values after NAR and Rg3 binding. (**D**) Solvent-accessible surface area after NAR and Rg3 binding. (**E**) Tyndall effect image of GNNs. (**F**) DLS particle size of GNNs. (**G**) TEM of GNNs. Scale bar: 100 nm. (**H**) Zeta potential of NAR, Rg3 and GNN. (**I**) UV–visible absorption spectrum. (**J**) FT-IR spectrum. (**K**) Release rate of GNN in FBS and SNF within 48 h. (**L**) Particle size and PDI change curves of GNN.

Drug molecule self-assembly was induced by controlling solvent polarity differences, effectively improving the drug’s solubility, stability, and bioavailability. Molecular dynamics simulation results showed that during a 100 ns simulation period, Rg3 and NAR can form stable complexes through hydrogen bonds and various non-covalent interactions (Figure 1A). Notably, 10–30 hydrogen bonds were continuously maintained between the two molecules (Figure 1B). This provided important support for the stability of the complex. Further analysis showed that the RMSD of the complex stabilized at around 4 nm after 50 ns (Figure 1C). This result suggests that the system has reached a conformational equilibrium state. Meanwhile, the SASA showed a gradually decreasing trend and stabilized after 50 ns (Figure 1D). This phenomenon further predicted that the complex formed a stable structure through the self-assembly process. The specific experimental procedure was that Nar and Rg3 were dissolved in 95% ethanol to form an organic phase, which was then uniformly injected into deionized water under ultrasonic conditions. During this process, solvent diffusion effects and hydrophobic interactions synergistically drove the self-assembly of drug molecules, ultimately forming nanoparticles with a core–shell structure (Figure 1E). DLS analysis showed that the average hydrodynamic diameter of the GNNs was around 100 nm, with uniform particle size distribution (Figure 1F); TEM images visually displayed spherical morphology, with size distribution highly consistent with DLS. (Figure 1G). Meanwhile, the nanoparticles exhibited high negative surface charge (−30.7 mV), which, combined with good monodispersity, ensured the stability of the colloidal system. (Figure 1H). FTIR spectroscopic analysis results showed (Figure 1I) that Rg3 exhibited a characteristic absorption peak at 1640 cm^−1^, corresponding to C=O stretching vibration; Nar also displayed corresponding characteristic peaks in this region. In the GNNs, these characteristic peaks underwent significant frequency shifts and intensity changes, with the peak near 1640 cm^−1^ shifting toward lower wavenumbers to approximately 1580 cm^−1^, while the peak shape also changed. We used UV–visible spectroscopy (Figure 1J) to investigate the GNNs. The UV spectrum of the GNNs showed a new absorption peak at 190 nm, which further corroborated the interaction between Rg3 and naringenin as well as the successful formation of nanoparticles (Figure 1K). The in vitro release curve indicated that the drug exhibited sustained release in simulated blood (pH 7.4, containing 10% FBS) and nasal fluid environments, with slower release in nasal fluid, which meets the sustained-release requirements for nasal delivery. Long-term stability studies showed that after storage at 4 °C for 7 days, GNNs maintained a particle size change rate of <5% and PDI < 0.25 (Figure 1). These indicate good formulation stability, meeting the basic requirements for clinical application.

### 2.2. GNNs Inhibit Aβ_1–42_-Induced Damage to Mouse Hippocampal Neurons

**Figure 2:**
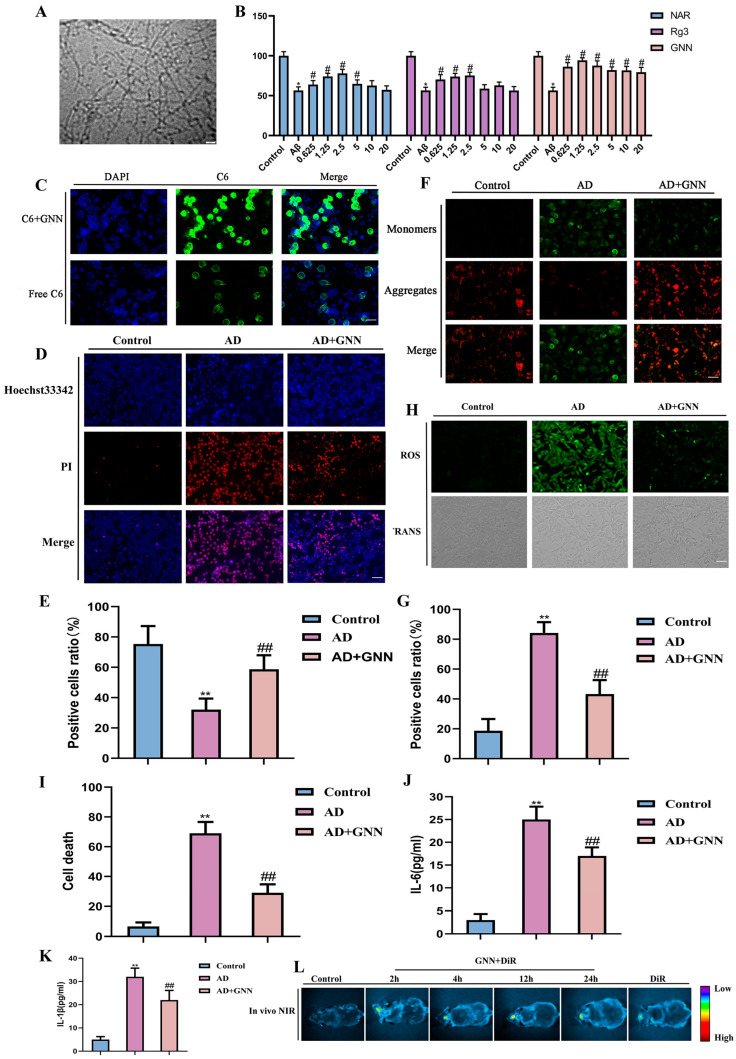
Therapeutic effects of GNNs on mouse hippocampal neuronal cells: (**A**) Electron microscopy results of Aβ_1–42_ oligomers. (**B**) CCK8 detection results of mouse neuronal HT22 cells. (**C**) Detection of C6 cellular uptake rate (green fluorescence). Scale bar: 150μm. (**D**) Double staining results of mouse neuronal cells with Hoechst33342/PI (blue: Hoechst33342, red: PI). Scale bar: 150μm. (**E**) Quantification of Hoechst33342/PI fluorescence. (**F**) JC-1 fluorescence staining results of mouse neuronal cells (green: J-aggregate, red: J-monomer). Scale bar: 150 μm. (**G**) Quantification results of JC-1 fluorescence. (**H**) ROS fluorescence staining results of mouse neuronal HT22 cells (green: ROS signal). Scale bar: 150 μm. (**I**) Quantification results of ROS fluorescence. (**J**) IL-6. (**K**) IL-1β. (**L**) In vivo real-time imaging. *, *p* < 0.05 vs. Control; **, *p* < 0.01 vs. Control; #, *p* < 0.05 vs. Model; ##, *p* < 0.01 vs. Model.

High-resolution TEM imaging was done to look at the ultrastructure of Aβ_1–42_ oligomers (Figure 2A). There is a fibrillar oligomer assembly structure with various electron densities. Most of the observed particles were in the size range of 15–40 nm, which corresponds to the typical toxic Aβ_1–42_ oligomer size, and this corresponds to the formation of the prefibrillar oligomeric intermediates thought to be involved in the pathogenesis of Alzheimer’s. To assess the protective effect of GNNs on Aβ_1–42_-induced harm to mouse hippocampal neurons, we induced HT22 cell injury in vitro by using Aβ_1–42_ oligomers (30 μmol) for 24 h, establishing blank, model, and treatment groups (0.3125 μmol, 0.625 μmol, 1.25 μmol, 2.5 μmol, 5 μmol, and 10 μmol). After 48 h, CCK-8 cytotoxicity testing showed that compared to the model group, all GNN treatment concentrations demonstrated significant neuronal therapeutic activity, significantly superior to NAR and Rg3 administered individually (Figure 2B). To further evaluate the cellular uptake capacity of GNNs, C6 was used as a fluorescent tracer in this study. The results showed that the GNN group exhibited strong and uniform green fluorescence distribution in HT22 cells, suggesting that GNNs have good cellular uptake capacity (Figure 2C). Hoechst33342/PI double staining results showed that, compared to the model group, cells in the GNN treatment group had more intact nuclear morphology and significantly fewer necrotic cells (Figure 2D,E). These results further indicate that GNNs have superior bioactivity and utilization compared to NAR and Rg3. As key factors in the AD pathological process, oxidative stress and neuroinflammation involve Aβ_1–42_, inducing ROS production and activating glial cells to release inflammatory factors, which further promote ROS generation, forming a “ROS–neuroinflammation” vicious cycle that continuously exacerbates neuronal damage. Based on this, this study further examined the oxidative stress and inflammation levels in HT22 cells. The results showed that Aβ_1–42_ treatment significantly increased intracellular ROS and inflammation levels, further leading to loss of mitochondrial membrane potential, while after GNN intervention, intracellular ROS generation and inflammatory responses were significantly inhibited, and mitochondrial membrane potential levels were restored (Figure 2F–K). GNNs labeled with markers were administered into the brains of C57 mice, and the changes in DiR signals were measured at 2, 4, 8, and 24 h (Figure 2L). At 24 h after administration, significant changes were observed, with fluorescence signals detected in the GNN DiR group, indicating that GNN DiR has excellent brain distribution. Meanwhile, free DiR also showed similar effects, while DiR-labeled GNNs exhibited higher accumulation in vivo, suggesting that these substances exert their effects through brain distribution. The above results confirm that GNN not only has good bioavailability and efficient cellular uptake capacity but also inhibits Aβ_1–42_-induced neuronal damage by blocking the “ROS–neuroinflammation” vicious cycle, providing important experimental evidence for nanomedicine treatment of AD.

### 2.3. GNNs Improve Cognitive Dysfunction Induced by Aβ_1–42_ Oligomers in Early AD Mice

To validate that GNN’s cognitive improvements result from reduced Aβ_1–42_ oligomer levels rather than non-specific nanoparticle effects, we conducted comprehensive Morris water maze assessments. Model group mice exhibited significantly higher escape latency during the training phase (5 days, 4 trials/day) compared to the control group (*p* < 0.01), indicating impaired spatial learning. In contrast, GNN treatment significantly reduced escape latency (*p* < 0.05 vs. model group), demonstrating improved learning acquisition.

During the probe trial (day 28), model-group mice spent substantially less time in the target quadrant (*p* < 0.01) and made fewer platform site crossings (*p* < 0.01) compared to the control group, reflecting deficient spatial memory. Conversely, GNN-treated mice spent significantly more time in the target quadrant (*p* < 0.05 vs. model group) and made more platform site passages (*p* < 0.05 vs. model group), indicating restored spatial memory consolidation.

**Figure 3:**
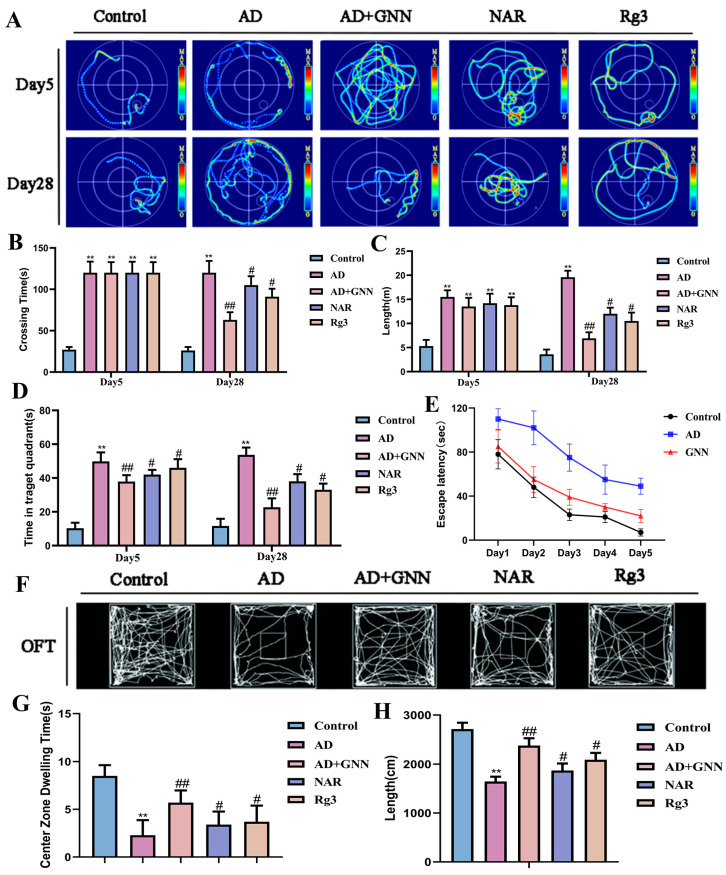
GNNs raise cognitive power in AD field mice: Morris water maze (MWM), open field test (OFT), early stage AD mice treated with GNNs, and late-stage AD mice treated with GNN. (**A**) shows the mice’s swimming trajectories; (**B**) the mice’s escape latency; (**C**) the total swimming distance of the mice; (**D**) the total time to find the platform; (**E**) time of the target zone of each group of mice; (**F**) the motion trajectory of mice in OFT; (**G**) total distance; (**H**) time spent in the target quadrant by mice. **, *p* < 0.01 vs. Control; #, *p* < 0.05 vs. Model; ##, *p* < 0.01 vs. Model.

Critically, GNN co-delivery demonstrated synergistically superior cognitive performance compared to equivalent doses of single-compound Rg3 monotherapy or NAR monotherapy (Figure 3A–E), supporting the hypothesis that Aβ_1–42_ reduction mediates the observed cognitive improvements through pathway-specific neuroprotection. In order to test the ameliorating effects of GNNs for anxiety-like behavior and autonomous behavior caused by Aβ_1–42_ oligomers, we performed the open field test (OFT). From the movement trajectory, it can be seen that the model group of mice tends to have a more chaotic pattern compared to the control group, which implies a decrease in exploratory ability (Figure 3F). Regarding the stay time analysis of the central area, in the model group, the mice spent much less time in the central area compared to the control group (*p* < 0.01), which is a very clear sign of being anxious (Figure 3G). After being processed by GNNs, the mice spent much more time at the center part (*p* < 0.05), which implies that their anxiety-like behavior was greatly mitigated. In terms of total movement distance, compared with the control group, the model-group mice had significantly lower total distances traveled (*p* < 0.01) and decreased ability to engage in activities of daily living (Figure 3H). GNN treatment greatly increased the total moving distance of the mice (*p* < 0.05 vs. model group) and improved the motor function of the mice effectively. It can be seen that compared with the NAR and Rg3 monomer groups at the same dosage, the GNN administration group displayed greater improvements in autonomous activity and reductions in anxiety-like behavior of AD mice. The result shows that GNNs can lessen the behavioral problems caused by Aβ_1–42_ oligomers.

### 2.4. GNNs Ameliorate Neuronal Apoptosis Induced by Aβ_1–42_ Deposition

**Figure 4:**
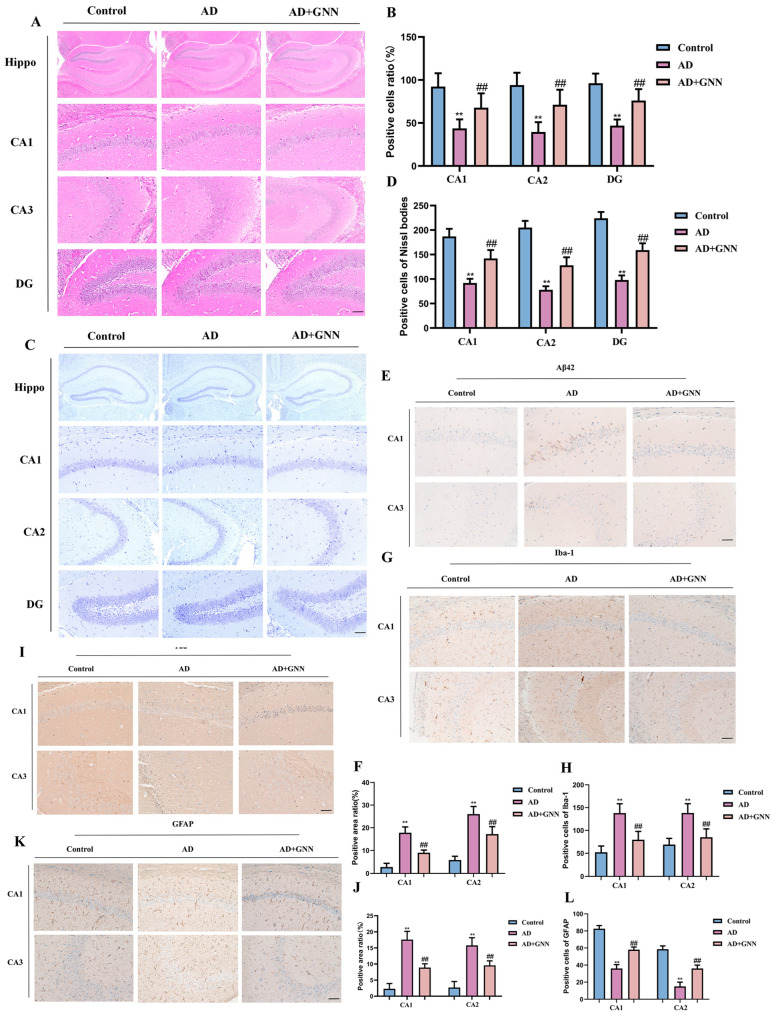
GNNs improve neuronal apoptosis in the brains of AD mice: (**A**) HE staining images of CA1, CA2, and DG regions in mouse hippocampus. Scale bar: 150 μm. (**B**) Percentage of healthy cells. (**C**) Nissl staining images of CA1, CA2, and DG regions in mouse hippocampus. Scale bar: 150 μm. (**D**) Number of healthy Nissl bodies. (**E**) Aβ immunohistochemical staining images. Scale bar: 150 μm. (**F**) Quantitative analysis of Aβ_1–42_ immunohistochemical staining. (**G**) Iba-1 immunohistochemical staining images. Scale bar: 150 μm. (**H**) Quantitative analysis of Iba-1 immunohistochemical staining. (**I**) Tau immunohistochemical staining images. Scale bar: 150 μm. (**J**) Quantitative analysis of Tau immunohistochemical staining. (**K**) GFAP immunohistochemical staining images. Scale bar: 150 μm. (**L**) Quantitative analysis of GFAP immunohistochemical staining. **, *p* < 0.01 vs. Control; ##, *p* < 0.01 vs. Model.

Brain tissue pathological analysis results showed that GNNs could significantly improve neuropathological changes in AD model mice. The control group showed that the neurons in the hippocampal regions (CA1, CA2, and DG) were neatly arranged and the cells were intact. Compared to the control group, the model group had a smaller quantity of neurons (*p* < 0.01); the neurons were in a loose irregular form, and there was a large amount of vacuole formation. However, upon treatment (30 mg/kg), the neuronal morphological changes were significantly alleviated, and the neuron numbers were significantly increased compared to the model group (*p* < 0.05) (Figure 4A,B). Nissl staining results showed that after treatment, the Nissl body density is significantly higher than that of the model group (*p* < 0.0). This indicates that GNNs are beneficial to neuron structure and function. The Nissl body is shown in Figure 4C,D.

The glial cell anomaly from the brain samples of the model mice was discovered; the immunochemistry test result was held back by GNNs. Compared with the control group, a large number of GFAP-positive cells and high staining intensity were observed in the brain tissue of the model mice (*p* < 0.001). This shows that there is abnormal activation of astrocytes. After the GNNs were processed, the number of GFAP-positive cells, as well as staining intensity, was markedly less in comparison to the model group (*p* < 0.01). This shows that the nanoformulation was able to curb overactive glial cells. Similarly, in the Iba-1 immunohistochemical staining experiment, the results were obtained by comparing the control group, and it was observed that the number of Iba-1-positive cells was significantly higher in the brain tissues of AD mice (*p* < 0.001), the staining was much stronger (*p* < 0.001), and the cells had the classic microglia activation morphology, such as a larger cell body and shorter processes. After GNN treatment, the number of Iba-1 cells and staining intensity were remarkably smaller compared with the model group (*p* < 0.01). This implies that GNNs can efficiently prevent microglia from pathological activity (Figure 4K,L). In summary, GNNs effectively alleviate brain tissue pathological changes in AD model mice, inhibit abnormal activation of glial cells, promote Aβ_1–42_ clearance, and exert neuroprotective functions.

### 2.5. GNNs Alleviate Neuroinflammation, Oxidative Stress Damage, and Protect Blood–Brain Barrier Function in AD Mice

**Figure 5:**
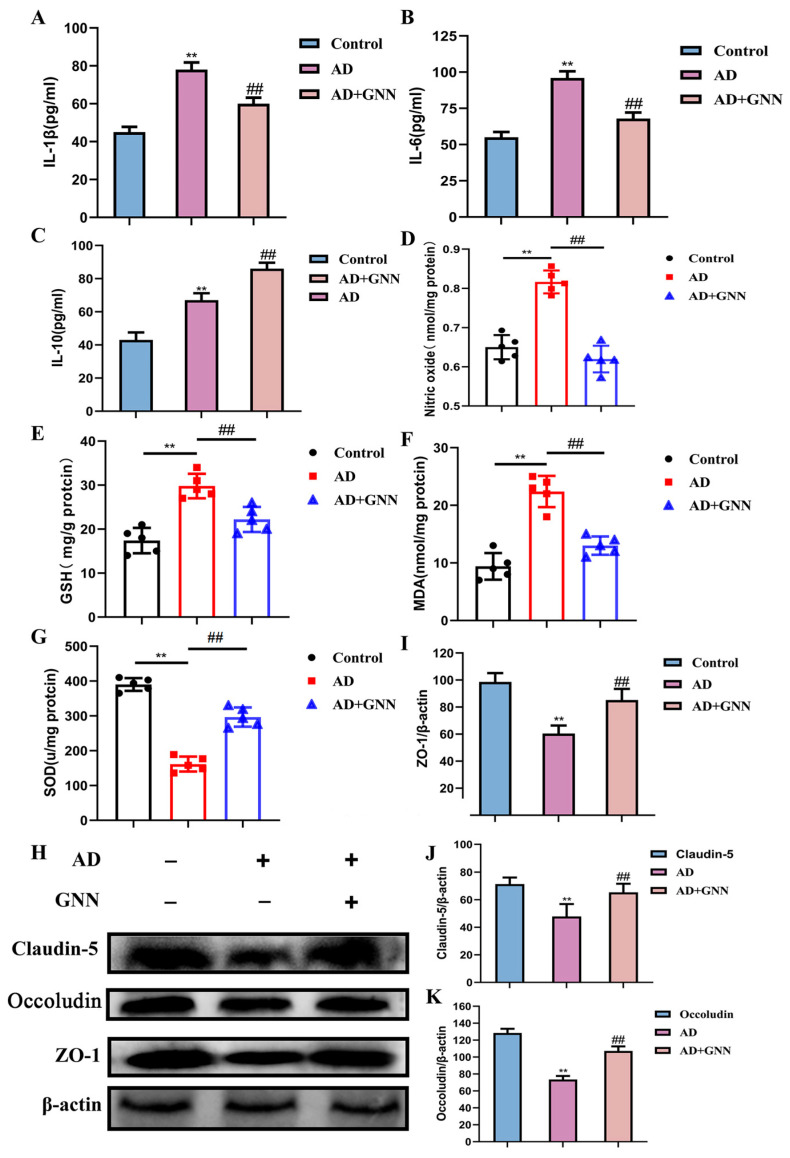
GNNs increase inflammatory factors IL-1β and IL-6, and the oxidative stress marker: (**A**) IL-1β, (**B**) IL-6, (**C**) IL-10, (**D**) NO, (**E**) GSH, (**F**) MDA, (**G**) SOD expression levels in AD mouse brains; they also maintain blood–brain barrier (BBB) integrity. (**H**) Representative immunoblots of Claudin-5, Occludin, and ZO-1, (**I**) quantified Claudin-5, (**J**) quantified Occludin, (**K**) Quantified ZO-1. **, *p* < 0.01 vs. Control; ##, *p* < 0.01 vs. Model.

In order to further prove in vivo activity of GNNs to break the vicious circle between Aβ_1–42_ and ROS-mediated neuroinflammation, inflammatory elements and oxidative stress-related factors of the mouse brain were also evaluated. About the inflammatory factor result, we found that the levels of IL-1β and IL-6 of the brain tissue of the GNN-treated mice were higher than the model group, whose levels decreased considerably, but the level of IL-10 of the group increased dramatically, which indicates that the GNNs can boost the inflammation factor expression of the mouse brain (*p* < 0.01) (Figure 5E–G). The oxidative stress-related index shows that the GNNs greatly improved the oxidative stress of AD model mice in the brain. In the result analysis, it is obvious that the drops in the NO level, GSH activity, and the SOD activity in the hippocampal tissue among AD model mice are quite significant compared to the control group (*p* < 0.01), as illustrated by Figure 5H–K, while the MDA content rose greatly (*p* < 0.01), which indicates that the brain tissue of AD model mice is severely damaged by oxidative stress. After GNN treatment, NO levels significantly increased (*p* < 0.05), GSH and SOD activities also significantly recovered (both *p* < 0.05), while oxidative damage product MDA levels significantly decreased (*p* < 0.05), indicating significantly improved antioxidant capacity. Aβ_1–42_ oligomers damage BBB integrity by inducing oxidative stress and neuroinflammation. Specifically, excessive ROS generated by Aβ_1–42_ cause endothelial cell oxidative damage, promote inflammatory responses, and directly downregulate tight junction protein expression, disrupting the BBB structure. To get the protective capability of the GNN BBB, we did Western blots on chief tight-junction proteins like ZO-1 and Occludin as well as Claudin-5 and evaluated them by looking at their function via the BBB permeability test. Results showed that Occludin, ZO-1, and Claudin-5 protein expression in AD mice were all less than in the control group (*p* < 0.01). After the GNN treatment, the change was reversed, and the levels of these three tight junction proteins were all well recovered in the AD + GNN group (*p* < 0.01). This shows that GNNs can keep the structure of the BBB unchanged.

### 2.6. Exploring the Mechanism of Action of GNNs in AD Mice Based on Transcriptomic Analysis

**Figure 6:**
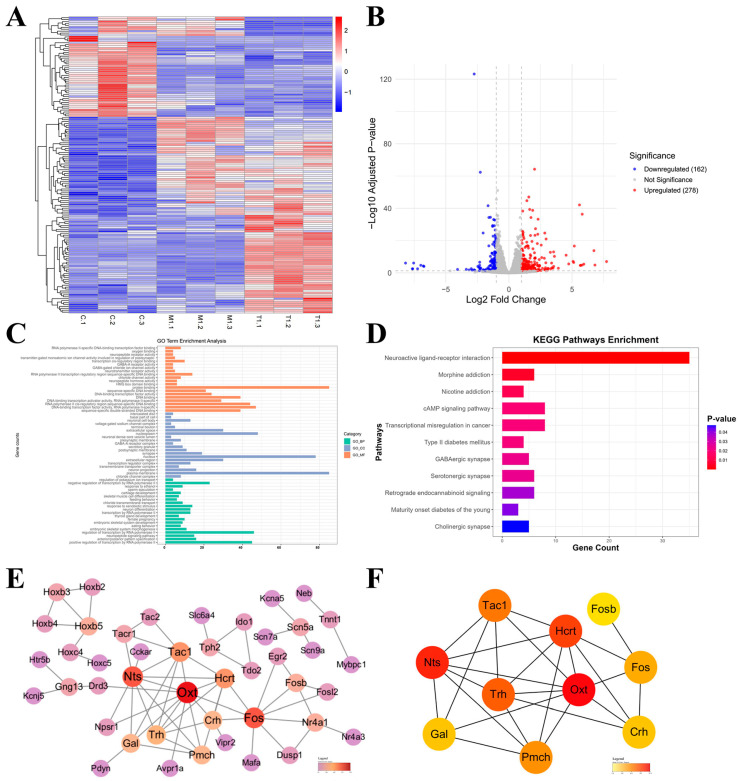
Transcriptomic analysis: (**A**) Heatmap. (**B**) Volcano plot. (**C**) KEGG. (**D**) GO. (**E**) PPI interaction network diagram. (**F**) Key gene network diagram.

In order to further explore the molecular mechanism of GNNs for treating AD, we did a transcriptome analysis of whole brain tissue from mouse before and after GNN treatment. The heatmap (Figure 6A) shows gene expression differences in the control group (C1–C3), model group (M1–M3) and treatment group (T1–T3). The results show good intergroup differences. Volcano plot (Figure 6B) analysis of the significantly differentially expressed genes revealed a total of 440 (278 upregulated, 162 downregulated, |log2FC| > 1, *p* < 0.05) genes.

KEGG pathway enrichment analysis (Figure 6C) showed that there was a significant increase in differentially expressed genes after all drug treatments, mainly in neuroactive ligand–receptor interaction pathways. GO for functional enrichment analysis (Figure 6D) showed that the different genes are mainly related to some bio-processes like neuronal cell bodies, synaptic plasticity, neural signaling transduction; they are also enriched in some synapse and neuronal membrane cellular components, and mainly function as some kind of neurotransmitter receptor involved in ion channel activity. The PPI network was generated by the string database and core regulatory genes were selected with the cyto hubba plugin of cytoscape. Analysis results (Figure 6E,F) further revealed key functional modules and core regulatory genes. A major protein interaction network was identified: a network dominated by acute phase proteins, including OXT, Nts, Hcrt, Pmch, Tac1, Fos, Gal, Trh, Crh, and Fosb. These genes are mainly involved in regulating the neuroendocrine system and central nervous system functions.

The above analysis results show that OXT and transcription factor Fos are core genes in the network and may be key targets for the drug’s efficacy.

### 2.7. GNNs Block the “ROS–Neuroinflammation” Vicious Cycle via the OXT/ERK/Fos Axis

**Figure 7:**
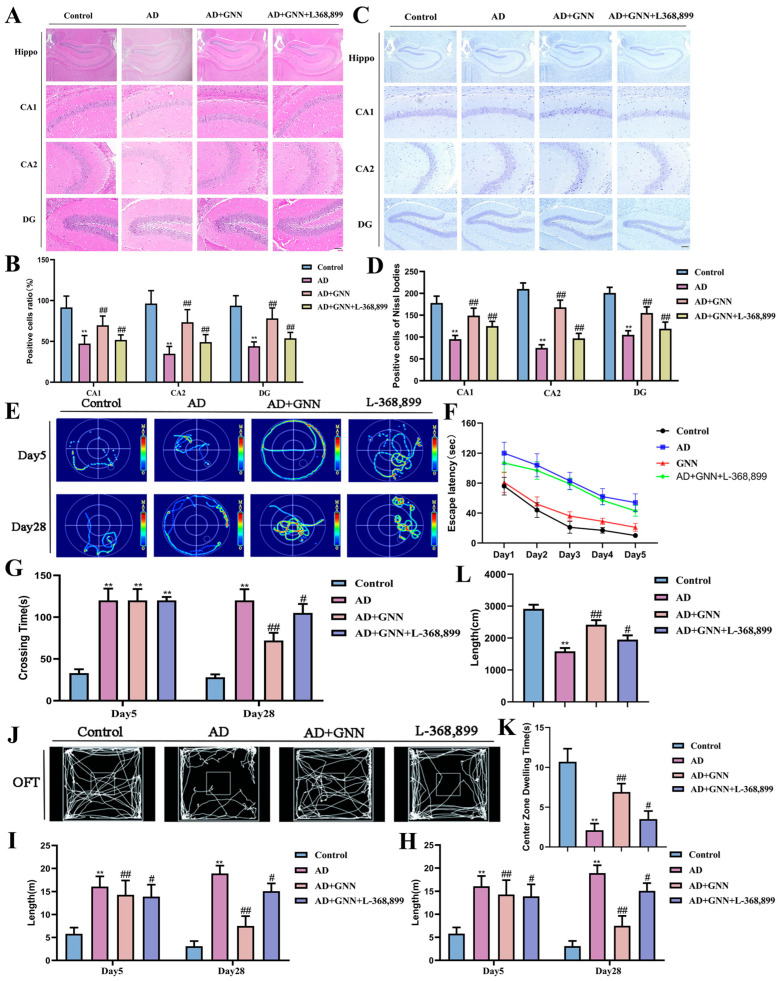
GNNs inhibit the ROS–neuroinflammation vicious cycle mechanism via the OXT/ERK/Fos axis: (**A**) Representative immunoblots showing expression levels of ERK, p-ERK, Aβ, Tau, and c-Fos. (**B**) Quantitative analysis of ERK and p-ERK proteins. (**C**) Quantitative analysis of Aβ protein. (**D**) Quantitative analysis of c-Fos protein. (**E**) Quantitative analysis of Tau. (**F**) IL-6. (**G**) IL-1β. (**H**) IL-10. (**I**) NO. (**J**) GSH. (**K**) MDA. (**L**) SOD. **, *p* < 0.01 vs. Control; #, *p* < 0.05 vs. Model; ##, *p* < 0.01 vs. Model.

Based on the transcriptomic study, we performed a Western blot on the protein levels of the OXT/ERK/FOS signaling pathway. As can be seen from the results (Figure 7A–E), it is obvious that compared with the control group, the content of OXT and c-Fos proteins and the p-ERK1/2/ERK1/2 level increased in the model group, and the change was more significant (*p* < 0.01), indicating that there were abnormalities in the activation of ERK signaling in the AD model. GNN treatment greatly enhanced OXT and c-Fos expression and curbed excessive ERK phosphorylation (*p* < 0.05). Pretreatment with OXT receptor antagonist L-368,899 blocked GNNs from having an effect on ERK phosphorylation and c-Fos expression, which shows that OXT is a key player in this pathway.

After pretreatment with OXT receptor inhibitor L-368,899, the effect of GNNs was reduced and anti-inflammatory and antioxidative effects were seen. Additionally, the expression of inflammatory factor expression levels was greatly increased (*p* < 0.05) and proved that protection occurred through the OXT/ERK/c-Fos signaling pathway.

The above results indicate that GNNs exert neuroprotective effects by activating the OXT/ERK/c-Fos axis to inhibit the vicious cycle of neuroinflammation and oxidative stress.

### 2.8. GNNs Exert Neuroprotective Effects by Inhibiting the ROS–Neuroinflammation Vicious Cycle via the OXT/ERK/Fos Axis

**Figure 8:**
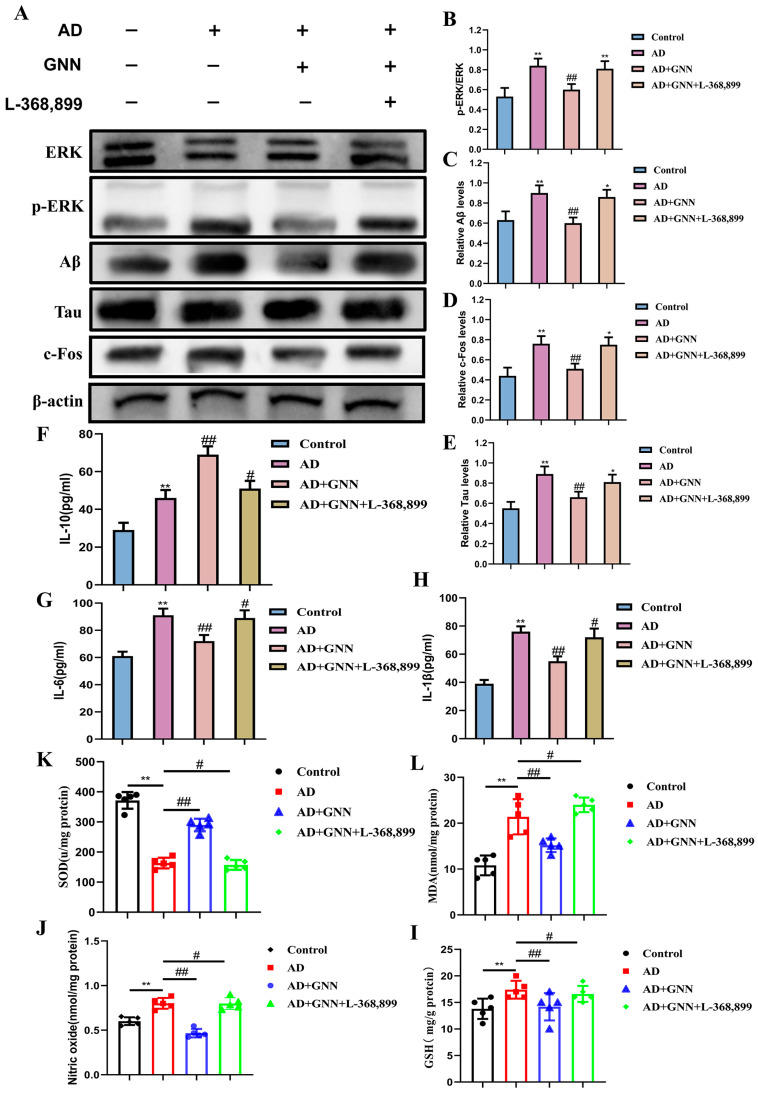
GNNs exert neuroprotective effects and cognitive improvement with OXT/ERK/Fos axis. (**A**) The HE staining picture of CA1, CA2, and DG areas of mouse hippocampus. Scale bar: 150 μm. (**B**) Percentage of healthy cells. (**C**) Nissl staining images of CA1, CA2 and DG regions in mouse hippocampus. Scale bar: 150 μm. (**D**) Number of healthy Nissl bodies. (**E**) The swimming trajectories of mice. (**F**) Escape latency. (**G**) Total time for all mice to find the platform. (**H**) Total swimming path of mice. (**I**) Time that the mice of each group spend in the target quadrant. (**J**) GNN evaluation on mice with OFT. (**K**) Total distance walked by all the mice. (**L**) Time spent by mice in target quadrant. *, *p* < 0.05 vs. Control; **, *p* < 0.01 vs. Control; #, *p* < 0.05 vs. Model; ##, *p* < 0.01 vs. Model.

To verify that GNNs exert therapeutic effects through the OXT receptor pathway, the OXT receptor-specific inhibitor L-368,899 was used in mechanism blocking experiments. Results showed that the OXT receptor inhibitor significantly reversed the therapeutic effects of GNNs. Histopathological examination indicated that L-368,899 pretreatment significantly weakened the protective effects of GNNs on hippocampal neurons, with improvements in neuronal survival rate and Nissl body density being blocked (*p* < 0.01) (Figure 8A–D). Behavioral test results were consistent, with L-368,899 treatment partially reversing the cognitive improvement effects of GNNs in the Morris water maze and open field tests (Figure 8E–L). These results indicate that GNNs exert neuroprotective and cognitive improvement effects by activating the OXT receptor signaling pathway, providing direct evidence for its mechanism in treating AD.

### 2.9. Biosafety Evaluation of Self-Assembled GNNs 

**Figure 9:**
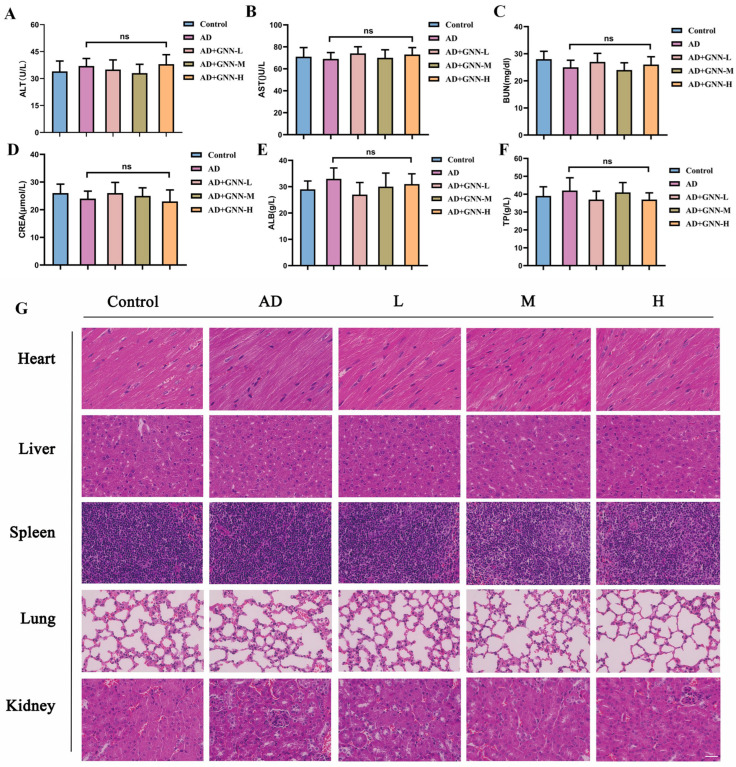
Biosafety evaluation of GNN (ns indicates no significant difference): (**A**) ALT. (**B**) AST. (**C**) BUN. (**D**) CREA. (**E**) ALB. (**F**) TP. (**G**) HE staining images of heart, liver, spleen, lung, and kidney. Scale bar: 150 μm. ns, no significant difference.

To comprehensively evaluate the biosafety and therapeutic effects of GNNs, this study detected serum biochemical indicators in experimental animals. Results showed no significant differences in biochemical parameters, including ALT, AST, BUN, CREA, ALB, and TP, between the treatment and control groups (Figure 9A–F) (*p* > 0.05). All indicators remained within normal physiological ranges, indicating that the dosing regimen in this study did not cause obvious liver or kidney toxicity or metabolic abnormalities. HE staining results proved that compared to the blank control group, vital organs such as the heart, liver, spleen, lung, and kidney of the treated group were kept normal in structure and cells, and had no pathological changes like inflammation infiltration, necrosis or fibrosis, which proved that the nano-delivery system has good compatibility with the organism within this experimental dosage (Figure 9G).

## 3. Discussion

Nano-delivery systems absolutely do have some pluses regarding drug bioavailability [28]. In this study, we chose ginsenosides Rg3 and Nar as active ingredients coming from nature, having good neuroprotective effects but with very limited clinical use because of low bioavailability [16]. We successfully achieved an GNN with an encapsulation rate of >80% and a particle size of (100 ± 10 nm), by using self-assembly nanotechnology. It means that its drug carrying capacity is sufficient. Because sustained release has to keep the same level of drug, DNPs could be taken in by cell(s) by Phagocytosis [29]. The results of our MD simulation show that the main cause for the creation of our self-assembled nanoparticle under this condition is the hydrogen bonding and hydrophobic interaction between Rg3 and Nar, based on the results of the previous research paper on self-assembled nanoparticles of natural products [30].

To verify the therapeutic mechanism of GNNs on Aβ_1–42_ oligomer-induced AD mice, this study established an AD model using Aβ_1–42_ oligomer-induced mouse neuronal cells. CCK8 detection showed optimal cell viability at 1.25 μg/ML concentration, which was determined as the optimal therapeutic concentration. The efficient cellular uptake observed through C6 fluorescence labeling indicates that our designed nano-delivery system successfully overcame cell membrane penetration barriers associated with traditional administration methods, significantly increasing intracellular concentration of active components, which may be an important foundation for its enhanced efficacy. More importantly, ROS staining results showed that GNNs significantly reduced Aβ_1–42_-induced intracellular oxidative stress levels; JC-1 detection confirmed their protective effect on mitochondrial function, and inflammatory factor detection results indicated that the nano formulation effectively inhibited Aβ_1–42_-induced neuroinflammatory responses. These in vitro experimental results are highly consistent with the antioxidant and anti-inflammatory effects we observed in vivo, further confirming the mechanism by which GNNs exert neuroprotective effects through synergistic regulation of the oxidative stress–inflammation axis.

To further verify the therapeutic effect of GNNs on Aβ_1–42_ deposition-induced AD mice, this study confirmed the significant effect of GNNs in improving Aβ_1–42_ oligomer-induced cognitive dysfunction through behavioral assessments. Morris water maze and open field test results showed that GNN treatment not only significantly shortened escape latency, and increased target quadrant dwelling time and platform crossing frequency, but also effectively alleviated anxiety-like behaviors, manifested as significant increases in central zone activity time and distance. More importantly, compared to using ginsenoside Rg3 or Nar alone, the dual-drug-loaded nanosystem demonstrated superior therapeutic effects in all cognitive function indicators, with escape latency further reduced by 30–40% compared to single-drug groups. These findings fully demonstrate the superiority of drug synergistic effects mediated by the nanocarrier system in neuroprotection, indicating that GNNs achieve synergistic effects by enhancing drug bioavailability, which is consistent with existing research results [31].

GNNs can effectively reduce brain tissue pathological changes in AD model mice, inhibit abnormal activation of glial cells, promote Aβ_1–42_ clearance, and exert neuroprotective functions. Oxidative stress and neuroinflammation are important aspects of AD. The continuous inflammatory reaction will make the neuron get hurt more quickly and the synapse work badly; it is a vicious circle for mutual promotion [32]. The Aβ_1–42_ oligomer causes an excess of ROS damage, MT dysfunction and oxidative damage to lipids, proteins and DNA [33]. Too much ROS will make more microglia and astrocytes to be active and release a lot more inflammatory factor, like IL-1β, IL-6, TNF-α and other chemokines [34]. These inflammatory mediators not only directly hurt nerve cells, but they also cause more ROS to be generated. A vicious circle is created: ROS–neuroinflammation, that is, no matter how many neurons are damaged, they are continuously aggravated by inflammatory cells and in a continuously amplified symbiosis process [35]. The results show that GNNs can break this vicious circle. On the other hand, their very strong antioxidant effect could recover SOD antioxidant enzyme activity of the hippocampus in AD mice, reduce MDA content and ROS quantity, basically reducing the damage of oxidation. On the other hand, due to suppressing the overly excited microglia (Iba-1) and astrocyte (GFAP) and so on, the expression of inflammatory factors such as IL1B and IL6 has been greatly reduced, thus blocking the inflammatory reaction amplification effect. Furthermore, GNNs can also produce anti-inflammatory factors like IL-10, so as to maintain immune balance and relieve neuroinflammation caused by Aβ deposition [36,37]. Through this dual regulation, GNNs can affect two important parts in this vicious circle. It can significantly reduce the pathological changes in brain tissues in the AD mice model. At the same time, it clears Aβ deposition and protects the nervous system with a strong neuroprotective effect. This is the same as the previous result about the anti-inflammatory and antioxidant effects of Rg3 and Nar [38,39].

To explore other nanomaterials that can be used to treat AD, the author used transcriptome sequencing to see what differentially expressed genes there are and also look at what enrichments can be found in these genes. The result was that it can also be seen that the differentially expressed genes were enriched in a neuroactive receptor–ligand pathway. PPI network analysis indicated that OXT and Fos may be the key genes mediating these effects. Among them, OXT is a neuropeptide that, besides participating in social behavior and emotional regulation, has recently been found to play important roles in cognitive function and neuroplasticity [40]. Fos, as an immediate early gene, has expression that can reflect the activation state of neurons and is closely related to learning and memory processes [41]. The oxytocin system has been reported to have antioxidant and anti-inflammatory properties [42]. Exogenous OXT has been proven to have therapeutic effects on AD [43]. Studies have also shown that oxytocin can alleviate oxidative stress damage by inhibiting the ERK pathway [44]. OXT activates the ERK signaling pathway through its receptor OXTR, and phosphorylated ERK1/2 then induces c-Fos expression, forming a complete signal cascade. Western blot analysis showed that Aβ_1–42_ oligomers promoted ERK phosphorylation and c-Fos expression, while GNN treatment significantly activated the OXT-ERK-Fos signaling pathway, characterized by upregulated OXT expression, restored ERK phosphorylation levels, and increased c-Fos expression. After treatment with the OXT inhibitor L-368,899, ERK phosphorylation levels and Fos expression were significantly reduced, and the neuroprotective effects and cognitive improvements of GNNs were significantly inhibited, confirming the key role of the OXT-ERK-Fos signaling pathway in the drug’s mechanism. This is consistent with previous research results on the role of ERK in neuroprotection [45]. Unlike traditional single-target drugs, GNNs may simultaneously regulate multiple pathological links through the OXT system, including neuroinflammation, synaptic plasticity, mitochondrial function, and BBB integrity. This multi-target synergistic effect may be the key to its significant improvement in cognitive function.

## 4. Materials and Methods

### 4.1. Materials

Ginsenoside Rg3 (purity > 98%), naringenin (purity > 95%), and coumarin-6 were all obtained from Yuanye biotech Co., Ltd., Shanghai, China. The HT22 cell line (C/L-0493) came from Sai Qi Biological Engineering Co., Ltd., Shanghai, China. Dulbecco’s Modified Eagle’s medium (DMEM), fetal bovine serum (FBS) and penicillin/strengthening were bought from Gibco Life Science Co. Ltd., Wyman Street, Waltham, MA, USA. The Cell Counting Kit-8 (CCK-8) and the Cell Apoptosis Hoechst 33342/PI Double Staining Kit were obtained from Solarbio Science &Technology Co., Ltd. Beijing, China. Aβ_1–42_ peptide was purchased from Macklin Biochemical Co., Ltd., Shanghai, China. The ROS detection kit was purchased from Beyotime Biotechnology Co. Ltd. (Shanghai, China) and the JC-1 detection kit was purchased from Beyotime Biotechnology Co. Ltd. (Shanghai, China). Primary antibody against ZO-1, Claudin-5, Occludin, p-ERK1/2, ERK1/2, c-Fos, Aβ and tau were all purchased from Proteintech Group, Wuhan, China and we labeled the horseradish peroxidase of the 2nd antibody as (PI Proteintech Group, Wuhan, China).

### 4.2. Preparation of Aβ_1–42_ Oligomers

The Aβ_1–42_ oligomer solution was prepared according to the method described by Bisceglia [46]. Prior to use, the Aβ_1–42_ stock solution was diluted with sterile PBS to form oligomers. Analysis was performed using transmission electron microscopy (TEM).

### 4.3. Molecular Dynamics Simulation

To understand the interactions between ginsenoside Rg3 and naringenin and their self-assembly behavior, molecular dynamics (MD) simulations were performed using the GROMACS 2020.4 software package (Groningen, The Netherlands) and the CHARMM36 all-atom force field [47]. A total of 10 Rg3 molecules and 10 naringenin molecules were randomly distributed in a cubic simulation box (3 × 3 × 3 nm^3^) and solvated with TIP3P water molecules. Long-range electrostatic interactions were calculated using the Particle Mesh Ewald (PME) method, and a cutoff distance of 1.0 nm was applied for van der Waals and short-range Coulomb interactions. Following energy minimization, the system was equilibrated under NVT (100 ps) and NPT (100 ps) conditions. A 100 ns production MD simulation was conducted with a 2 fs time step, and trajectories were analyzed for root-mean-square deviation (RMSD), hydrogen bonding, and solvent-accessible surface area (SASA) using standard GROMACS tools.

### 4.4. Preparation and Characterization of GNNs

We prepared GNNs using the solvent method. Ginsenoside Rg3 and naringenin at a 1:1 mass ratio (50 mg each) were dissolved in 10 mL of ethanol to form the organic phase. Under ice bath stirring conditions (600 rpm), the organic phase was slowly injected into 20 mL of deionised water. The rate of injection was 1 mL/min. This formed the aqueous phase. The mixture continued stirring for 1 h to allow partial ethanol evaporation and promote nanoparticle formation. The final dispersion was passed through the polyethersulfone (PES) membrane to remove unencapsulated drugs and aggregates, resulting in a clear GNN suspension [48].

A Malvern Zetasizer Nano ZS90 (Malvern Instruments Ltd., Malvern, Worcestershire, UK) was employed to ascertain the hydrodynamic diameter, polydispersity index (PDI) and Zeta potential of the particles via DLS. Morphology of the particles observed by transmission electron microscope (TEM) (JEOL JEM-1400, Tokyo, Japan). We used Fourier transform infrared spectroscopy (FTIR, PerkinElmer Spectrum 2, Shelton, CT, USA) to study the changes in chemical structure and intermolecular interaction of the nanoparticles. We used a UV-1900i spectrophotometer made by Shimadzu, Japan, to get the value of UV absorption spectra. HPLC (Agilent 1260, Santa Clara, CA, USA) was used to detect the encapsulation efficiency of Rg3 and naringenin. In vitro release behavior of the drug was identified by employing a dialysis bag in simulated physiological condition. We determined the drug content in the release medium with HPLC. Additionally, particle size and the PDI were used as stability indicators.

### 4.5. Cell Experiments

#### 4.5.1. Cell Culture

HT22 cells were cultured in high-glucose DMEM supplemented with 10% fetal bovine serum (FBS), 100 U/mL penicillin and 100 μg/mL streptomycin. Cells were maintained at 37 °C in a humidified incubator under 5% CO_2_ atmosphere. The culture medium was refreshed every 2–3 days to maintain optimal pH and nutrient levels. When cells reached 80–90% confluence, they were subcultured by trypsinization (0.25% trypsin-EDTA) and seeded into new culture flasks or experimental plates at appropriate densities.

#### 4.5.2. Cell Viability Assay

HT22 cells were treated with different concentrations of GNNs (1.25, 2.5, 5, 10, and 20 μg/mL). Subsequently, they were replaced with medium containing Aβ_1–42_ oligomers (30 μmol) for 24 h. Once fully processed, cell viability was detected using the CCK-8 assay.

#### 4.5.3. Detection of Cell Apoptosis Using Hoechst 33342/PI Apoptosis Detection Kit

HT22 cells were pretreated with different concentrations of GNNs. Following treatment, apoptotic cell morphology was evaluated using the Hoechst 33342/propidium iodide (PI) staining kit. Apoptotic cells exhibiting morphological characteristics such as chromosome condensation, nuclear fragmentation, and nuclear division were observed and quantitatively analyzed by fluorescence microscopy.

#### 4.5.4. Detection of ROS Levels

HT22 cells were pretreated with different concentrations of GNNs. ROS content was assessed using a ROS detection kit.

#### 4.5.5. JC-1 Assay

HT22 cells were pretreated with different concentrations of GNNs. Following co-incubation with the test compounds, cells were stained using a JC-1 detection kit.

#### 4.5.6. Determination of Cellular Uptake of GNNs Using Coumarin-6 Fluorescence Labeling Method

HT22 cells were treated with culture medium containing GNNs labeled with coumarin-6 fluorescent [49]. Once fully processed, the cellular uptake of the GNNs was observed using a fluorescence microscope [50].

#### 4.5.7. In Vivo Real-Time Imaging

Mice were administered GNN DiR intranasally (equivalent to a DiR dose of 0.1 mg/kg). After intranasal administration, in vivo imaging was performed using the IVIS Spectrum imaging system for small animals (PerkinElmer, Shelton, CT, USA). The system can be operated at specific time points. Imaging acquisition parameters: λex/em = 748/780 nm.

### 4.6. Experimental Animals and Grouping

In the International Scientific database, three-month-old male C57BL/6J mice (23 ± 1 g) were created by Vital River (Beijing, China) and acclimated at State Key Laboratory of Model Animals. The Mice were raised in animal-specific SPF-grade controlled living quarters and had free access to both food and water. All experiments with the animals were approved by the animal ethics committee of Jilin Agricultural University (Approval No. (2023-KJT-021) and followed the globally recognized standards for the treatment and utilization of laboratory animals. The mice were randomly divided into 6 groups, n = 8/group: (1) control group—infusion of same volume of saline into the lateral ventricle (SG); (2) model group—infusion of Aβ_1–42_ oligomer (LVA) into the lateral ventricle. (3) NAR group—LVA followed by intranasal NAR (50 mg/kg/day). (4) Rg3 group—LVA followed by intranasal Rg3 (50 mg/kg/day); (5) Aβ + GNN, AD + GNN—LVA followed by intranasal GNN (25, 50, 125 mg/kg/day); and (6) Aβ + GNN + L-368,899—L-368,899 (20 mg/kg/day, intraperitoneal injection) was administered in addition to GNN treatment. Treatment began on the 7th day after ventricular injection and lasted for 28 days.

### 4.7. Surgical Procedure and Drug Administration Protocol

Stereotaxic surgery was done as usual. We used a stereotaxic apparatus (RWD Life Science, Shenzhen, China). We fixed the mice under anesthesia with sodium pentobarbital (50 mg/kg, SP). According to in Stereotactic coordinates of the mouse brain by Paxinos and Franklin, the coordinates for the right lateral ventricles were determined [51]. A microinjection pump (RWD Life Science, Shenzhen, China)was used to inject 5 μL of Aβ_1–42_ oligomer solution or an equal volume of saline. The scalp incision was sutured and disinfected with povidone-iodine. Drug administration began 7 days after surgery. GNNs (30 mg/kg) were administered intranasally for 28 consecutive days [52]. L-368,899 was administered via intraperitoneal injection 30 min before GNN administration.

### 4.8. Behavioral Tests

The following behavioral tests were carried out after treatment to assess the cognitive function of the mice.

#### 4.8.1. Morris Water Maze Test

Morris water maze testing was carried out as described by Vorhees and Williams [53]. In summary, a 120 cm diameter circular water pool was divided into 4 pieces, with a constant temperature of 22 ± 1 °C. There was a clear plastic platform with a 10 cm diameter. It was fixed in one area. Training lasted for 5 days with 4 trials per day. Each mouse’s time to find the platform was noted down. We placed the mice into water from different quadrants for each run. A 15 s stay was allowed. Latency was recorded as 120 s. The starting position for each training trial was randomly selected. Experiments were carried out on days 5 and 28 of training. In these experiments, mice were put in the water in the quadrant that was opposite the one that they were aiming to reach. We timed how long it took to find the platform, how far they traveled before reaching it, and how long they spent in the platform quadrant. Data were recorded and analyzed using the ANY-maze behavioral analysis system (Stoelting Co., Wood Dale, IL, USA).

#### 4.8.2. Open Field Test

The apparatus comprised 4 open spaces each sized at 40 × 40 × 40 cm. Before starting the testing procedure, the mice were put inside the laboratory and allowed to adapt to the chamber for 1 h. Mice were placed in the center of the open field and allowed to move around freely for 10 min. The time in the center and movement speed were tracked via video (Shanghai Jiliang Software Technology Co., Ltd., Shanghai, China). The data was taken and analyzed with the ANY-maze behavior analysis system (Stoelting Co., Wood Dale, IL, USA).

### 4.9. Collection and Processing of Brain Tissue Samples

After behavioral testing, mice underwent deep anesthesia with SP and cardiac perfusion. Blood was removed by saline perfusion; brain tissue was excised and fixed in tissue fixative for 24 h before dehydration in 30% sucrose solution. OCT was used for embedding the brain tissue. We used embedding medium (Sakura Finetek, Torrance, CA, USA) and performed tissue sectioning on a cryostat microtome (Leica CM1950, Leica Microsystems, Wetzlar, Germany). The sections were appropriate for subsequent experiments.

### 4.10. ELISA Assay

We used commercial ELISA kits (Mlbio, Shanghai, China) to measure the levels of soluble IL-1β, IL-10, and IL-6 proteins in the body, and measured the levels of NO, SOD, MAD, and GSH in the middle of the hippocampus. All samples were tested in 3 repeated datasets. Absorbance was measured with a Multiskan™ FC microplate photometer (Thermo Fisher Scientific, Waltham, MA, USA), and the concentrations of all indicators in the sample were calculated according to the standard curve.

### 4.11. Western Blot

Total protein was extracted from brain tissue using RIPA lysis buffer and quantified by BCA assay. Protein samples (30–50 μg) were loaded onto SDS-PAGE gels (10–12%), transferred to PVDF membranes, and blocked with 5% non-fat milk for 1 h. Membranes were probed with primary antibodies against Claudin-5, Occludin, ZO-1, ERK, p-ERK, Aβ, Tau, c-Fos and β-actin (1:1000) overnight at 4 °C, followed by HRP-conjugated secondary antibodies (1:5000) for 1 h. Protein detection was performed using the enhanced Chemiluminescence kit (Merck Millipore, Burlington, MA, USA).and visualized with the ChemiDoc XRS+ system (Bio-Rad Laboratories, Hercules, CA, USA). Band intensities were quantified using Image J software version 1.53 (NIH, Bethesda, MD, USA) and normalized to β-actin.

### 4.12. Immunohistochemistry

Mouse brain tissue sections (10 μm) were prepared using a cryostat and mounted on glass slides. Sections were fixed in 4% paraformaldehyde, blocked with 5% normal goat serum, and incubated with primary antibodies against Aβ, Iba-1, Tau, and GFAP overnight at 4 °C. HRP-conjugated secondary antibodies were applied for 1 h at room temperature. Color development was performed using the DAB kit (Beyotime, Shanghai, China) followed by hematoxylin counterstaining. After dehydration through graded ethanol solutions and clearing in xylene, sections were mounted with neutral balsam. Images were captured using an optical microscope (Leica, Wetzlar, Germany) and analyzed with Image J software version 1.53 (NIH, Bethesda, MD, USA).

### 4.13. Statistical Analysis

All the data were shown as the mean plus or minus the standard error of the mean (SEM). Statistical analyses were done by using GraphPad Prism 8.0 software (GraphPad Software, USA). After performing a homogeneous variance test and normal distribution test, the data was analyzed using one-way analysis of variance (one-way ANOVA) for comparison among different groups, followed by Tukey’s test for multiple comparisons. The escape latency data of the water maze training period was subjected to two-way ANOVA and the Bonferroni post hoc test. The statistical significance level is as follows: *, *p* < 0.05; **, *p* < 0.01; ***, *p* < 0.001 (for comparisons vs. Control group); #, *p* < 0.05; ##, *p* < 0.01 (for comparisons vs. Model group); ns, no significant difference.

## 5. Conclusions

This study successfully constructed a nano-delivery system with ginsenoside Rg3 and Nar and verified its neuroprotective effects in an Aβ_1–42_ oligomer-induced AD model. Mechanism studies indicated that GNNs function by regulating the OXT-ERK-Fos signaling pathway, promoting OXT expression, reducing ERK phosphorylation levels and c-Fos expression patterns, effectively decreasing oxidative stress and neuroinflammation, improving Aβ metabolism, thereby significantly improving AD-related cognitive impairment. This study provides an innovative nano-delivery strategy based on the synergistic effects of natural products for early AD treatment.

## Data Availability

The data are contained within the article.

## Abbreviations

The following abbreviations are used in this manuscript:

AD	Alzheimer’s disease
BBB	Blood–brain barrier
Nar	Naringenin
GNN	Rg3-Nar-NP
ROS	Reactive oxygen species
OXT	Oxytocin
OXTR	Oxytocin receptor
MD	Molecular dynamics
RMSD	Root square deviation
SASA	Solvent-accessible surface area
PES	Polyethersulfone
PDI	Polydispersity index
DLS	Dynamic light scattering
FTIR	Fourier transform infrared spectroscopy
PI	Propidium iodide
LVA	Lateral ventricle injection of 5 μg Aβ_1–42_ oligomers
TEM	Transmission electron microscopy
C6	Coumarin-6
OFT	Open field test
Iba-1	Inhibiting the abnormal activation of microglia
PPI	Protein–protein interaction
TCM	Traditional Chinese medicine
DMEM	Dulbecco’s modified Eagle’s medium
FBS	Fetal bovine serum
CCK-8	Cell Counting Kit-8
